# Functional Connectivity Between Motor and Mid-Frontal Areas During Vicarious Reward Revealed via EEG Time-Frequency Analysis

**DOI:** 10.3389/fnhum.2019.00428

**Published:** 2019-12-04

**Authors:** Tsukasa Inomata, Takuro Zama, Sotaro Shimada

**Affiliations:** ^1^Electrical Engineering Program, Graduate School of Science and Technology, Meiji University, Kawasaki, Japan; ^2^Department of Electronics and Bioinformatics, School of Science and Technology, Meiji University, Kawasaki, Japan; ^3^Aoyama Gakuin University Research Institute, Tokyo, Japan

**Keywords:** EEG, phase synchronization, cross-frequency coupling, beta oscillatory activity, BOA, vicarious reward, vicarious experience

## Abstract

Vicarious reward is a phenomenon in which an individual feels as if he/she has received a reward as the result of watching someone else receive a reward. In this study, we used electroencephalography to investigate brain activity while participants watched a preferred player win a competitive game (Rock-Paper-Scissors game). In the experimental task, movie clips showed right hand of the two players and played Rock-Paper-Scissors game. We asked participants to explicitly support or “cheer” for a specific player, and then examined brain activity associated with vicarious reward. For the observed hand movement, previous findings showed that the event-related desynchronization of mu band (8–14 Hz) appeared at the contra-lateral central electrode to the observed hand (If someone sees the right-hand movement, the left central electrode shows the event-related desynchronization of mu-band). During observation of the player, we detected event-related desynchronization of mu band activity in the contra-lateral central electrode as well as mid-frontal beta band (15–22 Hz) activation when the preferred player won. Furthermore, functional connectivity analysis revealed a strong phase synchronization between the contra-lateral central electrode and mid-frontal electrode in the mu band when participants received the vicarious reward. Cross-frequency coupling analysis revealed functional integration between the mu and beta bands at mid-frontal electrode. These results indicate the interaction of mu band observed at contra-lateral electrode and beta band observed at mid-frontal electrode coupling, suggesting a link between the mirror neuron system and the reward system during vicarious reward.

## Introduction

Although humans are physically separated, we sometimes feel like we are psychologically connected to the people around us. For instance, our close friends may feel our pleasure as if it was their own. We may also feel pleasure when we observe someone that we know succeed at something or win a prize. The phenomenon by which we vicariously experience the sensations and emotions of others, which is called empathy, is important for social life. Most previous studies on empathy have focused on negative empathy, such as that associated with pain or suffering (Singer et al., 2004, 2006; Jackson et al., 2005, 2006; Bird et al., 2010; Hein et al., 2010; Lamm et al., 2011). However, empathy does not only refer to vicarious feelings of the pain of another individual but is also related to vicarious positive experiences and feelings of happiness. An increasing number of researchers are examining positive empathy (Lammel et al., 2008; Gable and Reis, 2010; Rizzolatti et al., 2014; Marco-Pallarés et al., 2015; Morelli et al., 2015; Apps et al., 2016). One form of positive empathy is vicarious reward, in which we experience rewards given to others as if we receive them ourselves (Lockwood, 2016).

In contrast to vicarious reward, many previous studies have investigated the experience of receiving a “direct” reward. Studies using functional magnetic resonance imaging (fMRI) have revealed that the ventral striatum (VS), ventromedial pre-frontal cortex (VMPFC), anterior cingulate cortex (ACC), and (in some cases) posterior cingulate cortex (PCC) are implicated in processing “direct” reward (Kelley, 2004; Rogers et al., 2004; Levy and Glimcher, 2011; Bartra et al., 2013; Häusler et al., 2015). For example, Rogers et al. (2004) reported that the ACC and VS were involved in the experience of a reward associated with a positive outcome for an individual in a gambling task (Rogers et al., 2004).

Brain activity during the experience of a “direct” reward has been investigated via electroencephalography (EEG). When participants experienced a monetary gain in a gambling task, beta oscillatory activity (BOA) was identified in the medial frontal region (typically around Fz, FCz, and Cz, Marco-Pallarés et al., 2008, 2015; Mas-Herrero et al., 2015). Marco-Pallarés et al. (2008) conducted a study using a gambling task in which valence (reward/monetary gain or punishment/monetary loss) and correctness (correct or incorrect choice) always coincided. They found an increase in beta power at the Fz electrode during gain vs. loss trials after the participants received feedback about the outcome of the trial (Marco-Pallarés et al., 2008). Mas-Herrero et al. (2015) conducted EEG and fMRI experiments using the same gambling task and fused the acquired data using Joint Independent Component Analysis (ICA) to investigate the neural network underlying BOA. They found that beta power increased after the participant received positive feedback. The fMRI analysis showed that the ventral striatum, caudate, ACC, and VMPFC were significantly activated in gain vs. loss trials. BOA in reward tasks is sensitive to the valence of events, such that outcomes that are better than expected elicit BOA while outcomes that are worse than expected do not (Marco-Pallarés et al., 2015).

In terms of vicarious reward, Shimada et al. (2016) revealed the activation of VMPFC when participants received the vicarious reward. In their study, participants watched a competitive game and while watching the game, they “cheered” for the particular player. After watching the game, they watched the same players participate in the stopwatch task. In the stopwatch task, the goal for the player was to press a button on a stopwatch so that the button press fell within ±0.05 s of the 5.00 s time point. When participants watched the “cheered-for” player at the competitive game succeeded the stopwatch task, VMPFC of the participants activated stronger than when they watched the failure of cheered-for player. Interestingly, opposite VMPFC activation pattern was observed for “non-cheered-for” player. VMPFC activation was larger than when participants watched the failure of non-cheered-for player and small VMPFC activation was observed when participants watched them succeed at the game. These results suggest that vicarious reward is processed in the VMPFC, which is activated specifically by the success of the other person with whom the individual feels unity or closeness. Another study by Mobbs et al. (2009) also support for this idea. They conducted an fMRI experiment in which participants watched a socially desirable player (SD) and a socially undesirable player (SU) play a game where they made a judgment about whether an unseen card would be higher or lower than a second unseen card. After the experiment, they asked participants to use a 10-point Likert scale to indicate their subjective responses in relation to the task. Subjective ratings acquired from the scale showed that participants perceived themselves to be more similar to, and in agreement with, the SD contestant. They found significant increase in ventral striatum (VS) activity, a region also active when the participants themselves won while playing the game. They also correlated perceived similarity scores of Likert scale for the SD > SU contestant win, which resulted in elevated VMPFC and ventral ACC activity. These studies suggest that although vicarious reward is processed in the same regions of the “direct” reward, it is affected by the subjective feeling to the observed player.

Previous studies reported that vicarious reward processing is affected not only by psychological factors that concern the observer, but also by other-oriented information. Apps et al. (2016) revealed that the ACC sub-region in the gyrus (ACCg) plays an important role in processing other-oriented information (Apps et al., 2013, 2016). Chang et al. (2013) used a modified dictator game in monkeys to show that the ACCg responded exclusively to rewarding outcomes delivered to others. These results indicate that interpreting the action of others is also important for vicarious reward processing.

Interpreting the actions of others in terms of their intentions and goals is essential to receiving vicarious rewards. The mirror neuron system (MNS) is thought to enable individuals to understand the actions of others (Rizzolatti et al., 2014). Functional neuroimaging studies have shown that during the observation and execution of actions, a cortical network is activated formed by the posterior part of the inferior frontal gyrus, the premotor cortex, and the inferior parietal lobe (Vanderwerta et al., 2012). In terms of EEG study, frequency between 8 and 14 Hz (mu) at electrodes corresponding to the sensorimotor regions of the brain (central electrode: typically, electrode sites C3, C1, Cz, C2, C4) was observed not only when performing an action but also when observing another person's action. When observing another person's action, desynchronization of the mu rhythm is observed (event-related desynchronization: ERD). The current standard method of detecting MNS activity using EEG is to investigate this mu rhythm at central electrodes (Arnstein et al., 2011; Braadbaart et al., 2013).

A recent study revealed that the MNS is also involved in the processing of vicarious rewards (Shimada et al., 2016). Shimada et al. (2016) used fMRI to examine the connectivity between the MNS and the reward system. In their study, participants explicitly supported or “cheered” for a particular player in a competitive or solitary game. The researchers found higher connectivity between the MNS and reward system when participants observed that the player was successful at the game.

To date, few studies have addressed EEG responses to vicarious rewards. Given the reports of BOA in the mid-frontal electrodes during reward processing and mu ERD in central electrodes during action observation (Arnstein et al., 2011; Braadbaart et al., 2013), we hypothesize that activity observed at the mid-frontal electrodes and central electrodes are reflected in change in power and that they occur during vicarious reward processing.

In this study, we conducted an EEG experiment to investigate the neural activity associated with vicarious reward. Specifically, we examined EEG activity that occurred when participants watched a competitive game (Rock-Paper-Scissors game: RPS game) and when they were asked to consciously support or “cheer” for a particular player, who then won the game. The experimental task comprised two sessions: the Action observation (AO) session and the Cheering (CH) session. In the AO session, participants were instructed to watch a single hand perform the RPS game (gesture), such that there was no winner or loser. This enabled us to examine MNS activity during action observation. In the CH session, the participants watched two hands compete at the RPS game, such that one hand won in each trial. They were instructed to cheer for a specific player to win the game. This enabled us to examine the EEG activity associated with vicarious reward processing and to investigate the functional connectivity between the MNS and the reward system while participants watched their preferred player win the game.

## Materials and Methods

### Participants

Twenty-two right-handed adults (1 female, aged 27.3 ± 8.9 years, mean ± SD) participated in the experiment. Six participants were excluded from the analysis because of a high amount of noise in the EEG signal (the noise detection method is described in the EEG data analysis section). A total of 16 participants were included in the analyses. Written informed consent was obtained from all participants. The protocol was approved by the Research Ethics Committee of Meiji University, and conducted according to the principles and guidelines of the Declaration of Helsinki.

### Experimental Tasks

**Figure 1:**
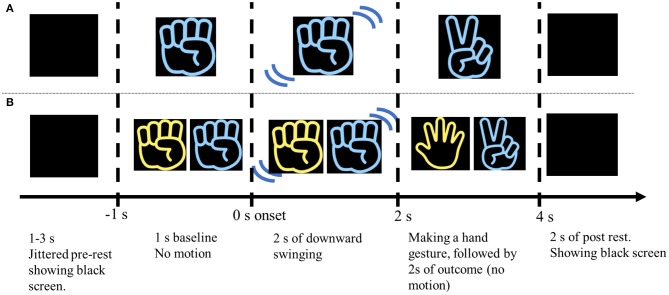
Schematic illustration of the experimental procedure in the AO and CH sessions. **(A)** The participants watched a 5-s movie clip of a RPS game performed by one (blue or yellow) right hand. The outcome gesture was apparent 2 s after the hand started moving. After the hand made a specific gesture, it remained motionless for 2 s, and then disappeared. The movie clip was followed by a 2 s black screen (post-rest period). Then the pre-rest period (jittered 1–3 s) began, which consisted of a black screen shown prior to the next movie clip. **(B)** The participants were instructed to consciously support or “cheer” for a specific colored hand (blue or yellow). The hand that the participant was instructed to “cheer” for always appeared on the right side of the screen. Winning and losing trials were presented in random order.

The experiment comprised two sessions: the Action Observation (AO) session and the Cheering (CH) session. Each session started with a 30-s rest period in which a computer display showed a black screen. In the AO session, participants were instructed to watch a movie clip in which one right-handed player performed the RPS game alone (Figure 1). Each trial consisted of a jittered pre-stimulus rest period (pre-rest, 1–3 s), a 5-s movie, and a post-stimulus rest period (post-rest, 2 s). In the movie clips in the AO session, the right hand appeared wearing either a blue or yellow glove. The color of the glove was randomized in each trial. After the pre-rest period, the right hand appeared on the screen and did not move for 1 s (baseline) before playing the RPS game. The hand performed a downward swinging motion two times for 2 s (action), and then formed a gesture symbolizing a rock, paper, or scissors (outcome). Thus, the delivery of feedback about the game outcome occurred 2 s after the hand started to move. After showing the outcome on the screen with the hand gesture, it stayed still and kept showing the hand gesture for 2 s so that the participants could easily understand which gesture is performed by the players. The hand gesture of the player in the movie clip was randomly selected in each trial. The sessions comprised three blocks, which each included 30 trials. The participant saw the blue- and yellow-gloved hands an equal number of times (45 times each). The purpose of the AO sessions was to investigate MNS activity while participants watched the RPS game without any feelings of allegiance toward the player, or any vicarious reward. The participants were instructed to simply watch the movie clip, and to maintain a neutral attitude about what they saw.

In the CH session, the participants were instructed to cheer for one of the two hands (blue or yellow) in the movie clip to win the RPS game (Figure 1B). They were asked to assume a situation like a close friend participate in the RPS game tournament, and cheer for the friend with all their heart. In the CH session, hands belonging to two players appeared on the screen and played the RPS game in each trial. The hand that the participants were asked to cheer for was always located on the right side. In the RPS game, rock defeats scissors, scissors defeats paper, and paper defeats rock. Situations in which both players made the same gesture (draw) were omitted because draw trials were not previously found to modulate specific activity related to vicarious reward (Shimada et al., 2016). As in the AO session, the movie clips were 5 s long in each trial and each block consisted of 30 trials. The sessions comprised 3 blocks, for a total of 90 trials. The blue- and yellow-gloved hands appeared an equal number of times (45 times). The number of trials in which the “cheered” for player won and lost was also equal, and the trials were presented in random order. The purpose of this session was to (1) investigate the neural mechanism of reward-processing associated with vicarious rewards, and (2) investigate whether the functional connectivity (phase synchronization) between the MNS and reward-processing areas occurs during vicarious reward. The CH session was conducted after the AO session so that the brain activity during action observation (e.g., MNS) was not affected by cheering.

### EEG Data Acquisition

EEG was recorded using a 24-bit bio-signal amplification unit (g.USBamp, g.tec Medical Engineering GmbH, Austria) with a sampling frequency of 1,200 Hz. The signals were recorded with active Ag/AgCl electrodes. Electrodes were mounted in an elastic cap and located at 30 positions according to the extended 10–20 system (Fp1, Fp2, F7, F3, Fz, F4, F8, FC5, FC3, FCz, FC4, FC6, T7, C3, Cz, C4, T8, CP5, CP1, CP2, CP6, P7, P3, Pz, P4, P8, PO3, PO4, O1, and O2). The ground electrode was located on the forehead and the reference was mounted on the right earlobe. Using the same amplification unit that we used to record EEG, we recorded vertical electro-oculography (EOG) from above and below the right eye. The electrophysiological signals were filtered with a 0.5–100 Hz band-pass filter.

### EEG Data Analysis

All analyses were performed in Matlab 2018a (Mathworks, Sherborn, MA, USA) software. We subjected the EEG data to Jade independent components analysis (ICA) to eliminate ocular artifacts. ICA components that were most significantly correlated with the vertical EOG (*r*^2^ > 0.16) were rejected. The remaining data were back-projected to create EEG signals that were free from ocular artifacts. After rejecting the ocular artifacts, we segmented the EEG data into 5-s (baseline 1 s, action 2 s, outcome 2 s) epochs. Outlier trials were excluded from analysis according to the following steps (Sanfey, 2007). (1) We selected the epochs in which EEG signals from more than eight electrodes (25% of the electrodes) exceeded ±3 standard deviations of the average amplitude at that epoch. (2) We set an artifact threshold of ±100 μV and excluded trials in which the signal exceeded this level from the analysis. (3) Among the selected trials, those that were visually recognized as outliers by the experimenter were excluded from further analysis. The percentage of rejected trials was 12 ± 9.6% (mean ± SD). The percentage of the rejected trials was not different between sessions [*t*_(15)_ = 0.40, *p* > 0.1 two-tailed *t*-test].

Prior to wavelet transformation, we re-referenced the EEG data, offline, to the mean of the amplitude from all electrodes. To investigate the time-frequency behavior elicited by the vicarious reward (Win/Lose), we computed the instantaneous amplitude and phase by convolving the EEG signal with a 6-cycle complex Morlet wavelet (Kajihara et al., 2015). The frequency ranged from 1 to 40 Hz in 1-Hz steps. We applied wavelet transformation to the entire set of noise-reduced EEG data that were pre-processed in the previous section. After wavelet transformation, the time-frequency data were normalized based on the power of each frequency during the first 30-s resting period. Then, data were baseline corrected using the average power of the baseline in each epoch (Hobson and Bishop, 2016). We avoided using the first half of the baseline period ([−1, −0.5] s) to reduce the influence of the previous trial. In the following statistical analysis, we used the normalized and baseline-corrected data to investigate the significance of power changes with respect to the different stimuli. We categorized the frequency bands as follows, (1) theta band: 4–7 Hz, (2) alpha band: 8–14 Hz, (3) low-beta band: 15–22 Hz, and (4) high-beta band: 23–35 Hz, and investigated whether there were any changes in the power of these bands when the participants received vicarious rewards.

### Mu ERD at C3 While Watching the RPS Game

In EEG research, mu ERD at the central electrodes had been widely hypothesized to represent MNS (Moorea et al., 2012; Cooper et al., 2013; Hobson and Bishop, 2017). Arnstein et al. (2011) simultaneously recorded EEG and BOLD-fMRI signals while participants observed and executed actions and found the regions that covaried with mu ERD were typically associated with the MNS. As previous findings showed that the mu ERD significantly appear to the contra-lateral region to the observed hand movement, we made a-priori selection of region of interest (ROI) of the electrode at C3 to investigate the mu ERD because the RPS game is played by right hand (Kajihara et al., 2015). To confirm the statistical significance of the mu ERD, we applied a paired sample two-tailed *t*-test using the average power of the baseline period and action period.

### Vicarious Reward Processing While Cheering for a Player

Previous studies have reported that BOA observed at the mid-frontal electrode signifies functional coupling of distributed brain regions involved in reward processing (Marco-Pallarés et al., 2008, 2015; Mas-Herrero et al., 2015), we made a-priori hypothesis of the ROI to the mid-frontal electrode (Fz, F4, FCz, and FC4) to investigate the vicarious reward processing. These electrodes are shown to reflect the BOA of reward processing by previous studies (Marco-Pallarés et al., 2008; Mas-Herrero et al., 2015). Normalized and baseline corrected data obtained from each electrode were tested using a two-way repeated measures ANOVA with two outcome conditions (Win/Lose) × three state periods (Baseline/Action/Outcome). We conducted an ANOVA for each of the five frequency bands mentioned above. We applied a threshold of *p* < 0.05 (corrected for family-wise error (FWE), for multiple comparisons across electrodes).

Our next goal was to confirm the current source of the frequency bands indicated by the ANOVA to be involved in vicarious reward processing. To this end, we estimated the current source during the outcome period using standardized low-resolution electromagnetic tomography (sLORETA) software (Pascual-Marqui et al., 2002). sLORETA is a popular method of EEG source localization and has been demonstrated by multiple studies to be efficacious (Lavric et al., 2001; Vitacco et al., 2002; Esslen et al., 2004; Herrmann et al., 2004; Szelenberger et al., 2005). Before applying sLORETA, we down-sampled the EEG data to 240 Hz to comply with the software restrictions.

### Phase Synchronization Index (PSI) and Cross Frequency Coupling (CFC)

To identify the phase relationships between any two electrodes, we defined the PSI for each time point and each electrode pair using the following equation (Kawasaki et al., 2010, 2014; Kajihara et al., 2015):

$$\begin{array}{l} PSI_{jk\left(t,f\right)}=\;\sqrt{{\left(\overset{N}{\underset{i\;=\;1}{\sum }}\frac{\cos\left(\Delta \phi_{jk}\left(i,f\right)\right)}{N}\right)}^{2}+\;{\left(\overset{N}{\underset{i\;=\;1}{\sum }}\frac{\sin\left(\Delta \phi_{jk}\left(i,f\right)\right)}{N}\right)}^{2\;}} \end{array}$$

where Δϕ_*jk*_(*t, f*) is the phase difference between electrodes j and k at time *t* and frequency *f*, and the number of time points *N* with an interval of 1.0 s is 1,200 (time window of [2.5, 3.5] s). To evaluate the vicarious reward-related PSI changes, we applied the bootstrap method to the PSI data. In the bootstrap method, the distribution of the statistic value under the hypothesis μ x = μ y (mean or median value of the two samples) is approximated using the bootstrap re-samples. To re-sample from a population that satisfies the null hypothesis, the original PSI data are converted to bootstrap re-sampled PSI data using the following formulas. Then, the re-sampled PSI data obtained during the outcome period [$\varphi_{jk}^{A^{*}}\left(t,f\right)$] are compared with those from the baseline period [$\varphi_{jk}^{B^{*}}\left(t,f\right)$]. Here, bootstrap re-sampled PSI data were calculated by the following equations:

$$\begin{array}{l} \varphi_{jk}^{A^{*}}\left(t,f\right)=\;\varphi_{jk}^{A}\left(t,f\right)-{\bar{\varphi }}_{jk}^{A}\left(t,f\right)+\;{\bar{\varphi }}_{jk}\left(f\right)\\ \varphi_{jk}^{B^{*}}\left(t,f\right)=\;\varphi_{jk}^{B}\left(t,f\right)-{\bar{\varphi }}_{jk}^{B}\left(t,f\right)+\;{\bar{\varphi }}_{jk}\left(f\right) \end{array}$$

**Figure 2:**
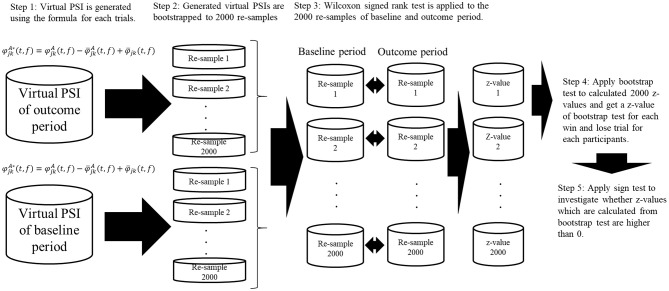
Schematic illustration of bootstrap procedure of phase synchronization index (PSI) and cross frequency coupling. Virtual PSIs are created by using the formula and 2,000 bootstrapped re-samples are created using the virtual PSIs for baseline period, outcome period for each trials of win and lose. After calculating the bootstrapped re-samples, non-parametric Wilcoxon signed-rank test was applied to the produced virtual PSIs and *z*-values are calculated. Then we applied bootstrap test to these *z*-values. After calculating the *z*-values from bootstrap test, we applied sign test to bootstrap test *z*-values to investigated whether they are significantly higher than 0.

Where $\varphi_{jk}^{A^{*}}\left(t,f\right)$ and $\varphi_{jk}^{B^{*}}\left(t,f\right)$ represent the re-sampled PSI. $\varphi_{jk}^{A}\left(t,f\right)$ and $\varphi_{jk}^{B}\left(t,f\right)$ represent the original PSI, and ${\bar{\varphi }}_{jk}^{A}\left(t,f\right)$, ${\bar{\varphi }}_{jk}^{B}\left(t,f\right),$ and ${\bar{\varphi }}_{jk}\left(f\right)$ represent the mean $\varphi_{jk}^{A}\left(t,f\right)$, mean $\varphi_{jk}^{B}\left(t,f\right)$, and the average value of all the data, respectively (Kawasaki et al., 2010, 2014). Using the non-parametric Wilcoxon signed-rank test for 2,000 bootstrapped re-samples for each time point for individual subjects, we calculated the *z*-values at which the PSI values during the outcome period were higher than those during the baseline period. For these *z*-values, we applied bootstrap test and calculated z-values of bootstrap test. For these bootstrap test *z*-values for individual participants, we applied a sign test between win trials and lose trials (Figure 2).

We also calculated the phase–phase cross-frequency coupling (CFC) between the alpha and beta oscillations at single electrodes (Kawasaki et al., 2010). We applied the PSI formula using Δϕ_*alpha*−*beta*_(*t, j*) to express the phase difference between two alpha phases (2 × ϕ_*alpha*_) and the beta phase (ϕ_*beta*_) at the j electrode because the relationship between the alpha and beta phases were expressed in the ratio 1:2 (e.g., beta have two peaks/troughs while alpha have one peak/trough).

The CFC was calculated using the equation:

$$\begin{array}{l} CFC_{alpha-beta}\left(t,j\right)=\\ \sqrt{{\left(\overset{N}{\underset{x\;=\;1}{\sum }}\frac{\cos\left(\Delta \phi_{alpha-beta}\left(x,j\right)\right)}{N}\right)}^{2}+{\left(\overset{N}{\underset{x\;=\;1}{\sum }}\frac{\sin\left(\Delta \phi_{alpha-beta}\left(x,j\right)\right)}{N}\right)}^{2}} \end{array}$$

$$\begin{array}{l} \Delta \phi_{alpha-beta}\left(x,j\right)=2\;\times \;\phi_{alpha}\left(x,j\right)-\;\phi_{beta}\left(x,j\right) \end{array}$$

If thinking the phase difference based on the number of peaks/troughs of frequency, and two frequencies are synchronized, alpha phase multiplied by 2 and beta phase will have same number of peaks/troughs and will take same phase. At this situation, the phase difference of alpha phase multiplied by 2 and beta phase will be smaller.

For these data, as with the within-frequency phase synchronization analyses, the CFCs for each trial were re-sampled and tested using the same procedure as for the PSIs. We calculated the CFCs to investigate the cross-frequency coupling, which are thought to represent different brain functions.

For the Wilcoxon signed-rank test and sign test, we applied a one-tailed test to investigate whether the PSIs and CFCs at the outcome were higher than those at baseline.

## Results

### Mu ERD at Central Electrodes During Action Observation

**Figure 3:**
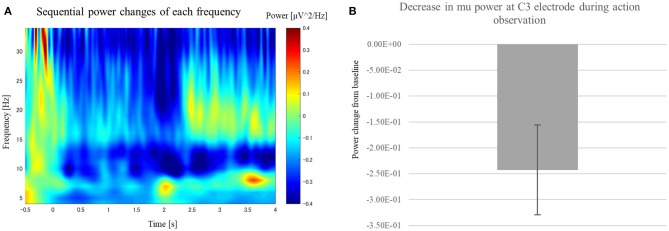
Change in power at the C3 electrode while participants watched the RPS game in the AO session. **(A)** Scalogram showing power at the C3 electrode while participants watched the RPS game in the AO session. **(B)** We observed significant power suppression at 8–14 Hz after the player began swinging their hand [*t*_(15)_ = 2.79, *p* < 0.05].

To identify mu ERD that occurred while the participants watched the RPS game, we applied time-frequency analysis to the motor-area electrode (C3) for the AO session. We found significantly greater mu ERD in the action period [paired sample two-tailed *t*-test, *t*_(15)_ = 2.79, *p* < 0.05] compared with that in the baseline period (Figure 3), indicating that the MNS was activated during action observation in the AO session.

### Reward-Related Power Increase for Vicarious Reward

**Figure 4:**
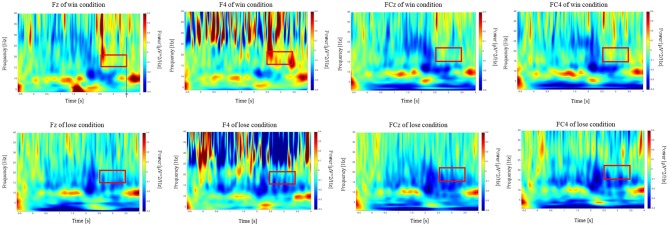
Scalogram showing power at the F4, FCz, and FC4 electrodes during the CH session in terms of frequency and time. Vertical axes show the frequency from 4 to 40 Hz. Horizontal axes show the time course of the trial. The power during the outcome period in the 15–22 Hz frequency range (red box) was higher in the win trials compared with that in the lose trials.

**Figure 5:**
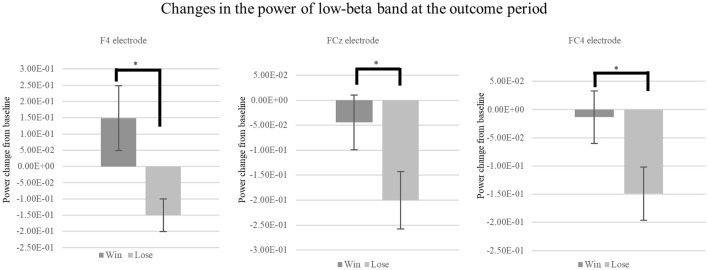
Mean change in the power of low-beta oscillations at outcome period during the CH session. We found a significant simple main effect between the low-beta oscillations in the Win and Lose trials during the Outcome period [F4: *F*_(1, 45)_ = 17.752, *p* < 0.05, FCz: *F*_(1, 45)_ = 18.859, *p* < 0.05, FC4: *F*_(1, 45)_ = 25.199, *p* < 0.05, FWER corrected]. *p < 0.05.

Time-frequency analysis of the outcome period showed a significant difference between the win and lose trials. Specifically, time-frequency analysis of the two conditions (Win/Lose) revealed greater power in the low-beta band at the frontal electrodes in the win condition compared with that in the lose condition during the outcome period (Figure 4). We used [2.5, 3.5] s as the time window for the outcome which showed largest power change from baseline irrespective of the outcome (win or lose). We conducted a two-way repeated measures ANOVA for the mean power change in the low-beta bands for the two outcome conditions (Win/Lose) × three state periods (Baseline/Action/Outcome). The ANOVA revealed a main effect of outcome at FC4 [*F*_(1, 15)_ = 12.132, *p* < 0.0167]. There is no significant effect at other electrodes [Fz: *F*_(1, 15)_ = 3.344, *p* > 0.05, F4: *F*_(1, 15)_ = 6.773, *p* > 0.05, FCz: *F*_(1, 15)_ = 7.475, *p* > 0.05]. It also revealed a significant main effect of period at FCz and FC4, but not at other electrodes [FCz: *F*_(2, 30)_ = 6.584, *p* < 0.05, FC4: *F*_(2, 30)_ = 5.717, *p* < 0.05, Fz: *F*_(2, 30)_ = 2.506, *p* > 0.05, and F4: *F*_(2, 30)_ = 1.157, *p* > 0.05]. It revealed a significant interaction between outcome × period in the low-beta band at F4, FCz, and FC4, but not at Fz [F4: *F*_(2, 30)_ = 5.607, *p* > 0.05, FCz: *F*_(2, 30)_ = 6.473, *p* < 0.05, FC4: *F*_(2, 30)_ = 7.535, *p* < 0.05, and Fz: *F*_(2, 30)_ = 3.383, *p* > 0.05]. Simple main effect analyses revealed a significant difference between the win and lose conditions in the outcome period [F4: *F*_(1, 45)_ = 17.752, *p* < 0.05, FCz: *F*_(1, 45)_ = 18.859, *p* < 0.05 FC4: *F*_(1, 45)_ = 25.199, *p* < 0.05] (Figure 5). Multiple comparisons at F4 revealed significant power enhancement during the outcome period compared with the action period [*t*_(60)_ = 2.321, *p* < 0.05] in the Win trials. Multiple comparisons at FCz revealed significant power suppression during the action period [*t*_(60)_ = 3.374, *p* < 0.05] and the outcome period [*t*_(60)_ = 4.374, *p* < 0.05] compared with the baseline period in the Lose trials. Multiple comparisons at FC4 also revealed significant power suppression during the action period [*t*_(60)_ = 3.510, *p* < 0.05] and the outcome period [*t*_(60)_ = 3.983, *p* < 0.05] compared with the baseline period in the Lose trials (FWER corrected between the electrodes comparison). In the high-beta band, there was neither a significant main effect nor an interaction.

**Figure 6:**
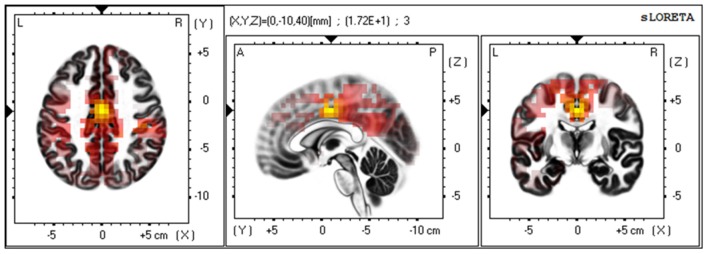
Current source estimation of the low-beta oscillation via LORETA. A group comparison of low beta-band current source density (CSD) for the point at which participants received the outcome of the RPS game (Win/Lose via *t*-test and SnPM randomization) revealed a statistically significant difference at the cingulate cortex [*t*_(15)_ = 17.18, *p* < 0.01. MNI coordinate: X = 0, Y = −10, Z = 40, BA24]. The yellow color indicates significantly higher levels of current density when the “cheered” for player won.

Subsequently, we investigated the current source of the low-beta band at the outcome period [2.5, 3.5] s using sLORETA. The result identified the posterior side of the anterior cingulate cortex (pACC) as a source region that exhibited significantly greater activation when participants watched the player they were cheering for win, as opposed to lose. This confirmed that the observed BOA originated from the medial pre-frontal area (Figure 6).

### Phase Synchronization Analyses

**Figure 7:**
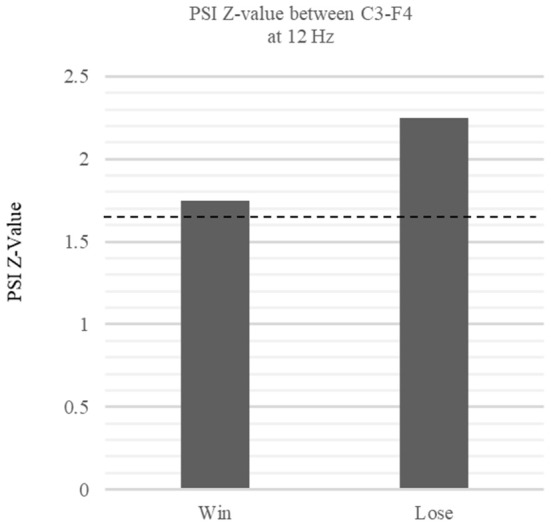
Comparison of PSI Z-values in win vs. lose trials between C3 and F4 phase relationship. The figure shows the *z*-values of the within-frequency phase synchronization index (PSI) between the win and lose trials at 12 Hz. The dotted lines denote the threshold value (*p* < 0.05, one-tail). The phase relationship between C3-F4 at 12 Hz exceeded the threshold during the win trials, indicating significant functional connectivity between C3 and F4 (Win: *Z* = 1.75 *p* < 0.05, Lose: *Z* = 2.25 *p* < 0.05).

According to the results of the time frequency analysis, we used C3 electrode which reflect MNS and the F4 electrode which showed significant power enhancement at win trials as reward-related electrode for further analyses. As formula of PSI needs to select specific frequency band, we choose 12 Hz in mu band which showed largest mu ERD at AO session. We found significant PSIs between C3 and F4 electrode in the mu band during win trials (*Z* = 1.75, *p* < 0.05) and lose trial (*Z* = 2.25, *p* < 0.05) (Figure 7). These results indicate that MNS is dynamically linked with a wide region in the mid-frontal electrode via mu ERD during the outcome period.

### Cross Frequency Coupling

**Figure 8:**
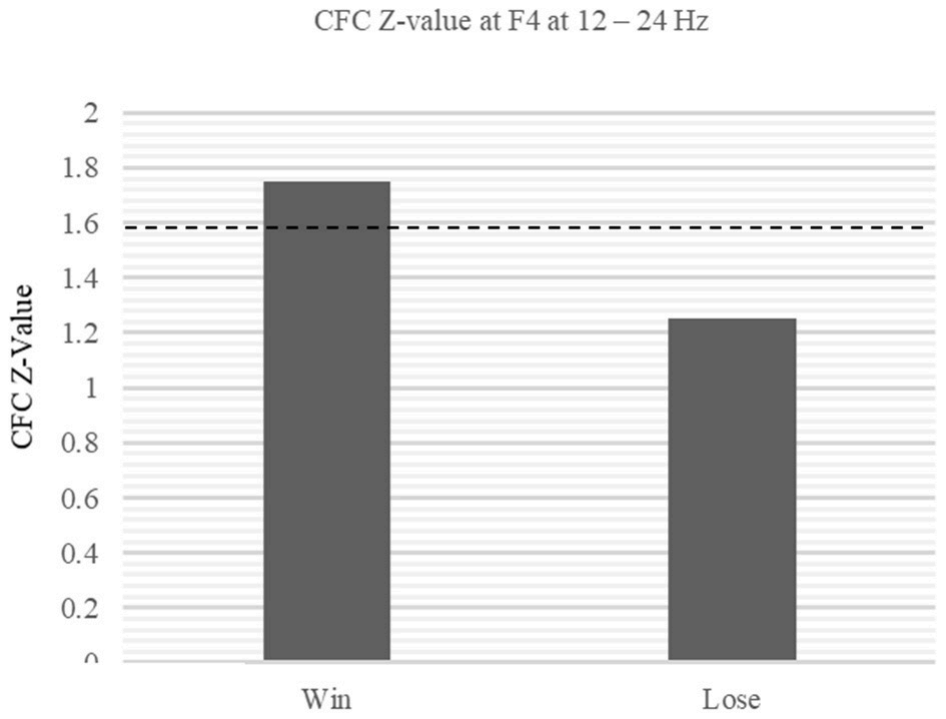
Within-electrode comparison of CFC *Z*-values between win and lose trials at F4. The *Z*-values of the CFC at F4 exceeded the threshold during win trial (Win: *Z* = 1.75, *p* < 0.05, Lose: *Z* = 1.25, *p* > 0.05 at 12–24 Hz).

Subsequent CFC revealed mu-beta synchronization at F4. We found significant CFCs during the win trials (Win: *Z* = 1.75, *p* < 0.05, Lose: *Z* = 1.25, *p* > 0.05 at 12–24 Hz; Figure 8). Such CFC implies the integration of MNS and reward information, which may be processed at the mu and beta frequencies, respectively.

## Discussion

Our experiment revealed BOA at the mid-frontal electrode during vicarious reward. The sLORETA analysis revealed that this BOA originated in the posterior side of the ACC. In addition, we observed mu ERD from the motor area electrode (C3) during action observation (AO session), which supposedly reflected MNS. Functional connectivity analyses revealed high phase synchrony between the mid-frontal electrode (F4) and the motor area electrode (C3) at 12 Hz during both the win and lose trials, indicating that mu band at C3 is dynamically linked with the F4 electrode which is located at mid-frontal area. Subsequent CFC analyses between the alpha and beta bands at the F4 electrode revealed higher phase synchrony between 12 and 24 Hz when the cheered for player won compared with when they lost. These results indicate interaction of the mu band and beta band at F4 electrode.

Previous studies have suggested that mid-frontal BOA occurs when an individual receives a reward (Marco-Pallarés et al., 2008, 2015; Mas-Herrero et al., 2015). In the current study, we observed slightly lower BOA that has been reported in previous research. These results are similar to the finding of van de Vijver et al. (2011) reported. Van de Vijver used a time estimation task whose feedback represents the accuracy of the response (correct or incorrect). The result showed slightly lower frequency (17–24 Hz) power increase at correct trial. In addition, this low-beta power was correlated to the next trial success of time estimation task. At their study, they concluded that the low-beta power was not only responsive to positive feedback but functioned as a learning signal as well. This is supported by another study which found beta desynchronization following error feedback in a time estimation task (Luft et al., 2014). Luft et al. (2014) also used a time estimation task and they found high-learner at time estimation task showed higher low-beta desynchronization at error feedback. These results can be hypothesized that this lower BOA reflects processes that related to mentally retrieving and correcting the previous trial (Luft, 2014). In current study, participants “cheered” for the specific player to win the RPS game. It seems reasonable that participants mentally retrieve and think about the result of previous trial after getting the outcome of the game. These results indicate that there are two distinct forms of BOA in response to outcome processing. The first is the BOA which responds to reward itself observed in many studies (Marco-Pallarés et al., 2008, 2015; Mas-Herrero et al., 2015). The second is the BOA which responds to mentally retrieving and correcting the previous trial.

The current source estimation using sLORETA revealed the source region of the BOA to be the middle of the cingulate cortex, which is located at BA24. As it is broadly located from the anterior side to the middle of the cingulate cortex, BA24 is often categorized as a part of the ACC (Apps et al., 2016). Previous studies have reported the involvement of the ACC in processing other-oriented information, including reward processing. The ACC also appears to process both positive and negative outcomes that are the result of decision-making by others (Leng and Zhou, 2010; Apps et al., 2013, 2016). Apps et al. (2013) reported that the anterior cingulate cortex gyrus (ACCg) modulates reward processing selectively when it is oriented toward others, and that it does not respond to rewards received by the self. Moreover, the posterior side of the ACC has been found to modulate positive outcomes that are the result of decisions made by others. Therefore, the ACC is thought to play a significant role in processing “other-oriented” information. These data appear to be consistent with our present findings because observation of the RPS game and the mental action of “cheering” for a player involve the processing of information that pertains to others.

With respect to connectivity analysis, previous studies have reported the involvement of the MNS in vicarious reward processing. Shimada et al. (2016) described the connectivity between the premotor cortex and vmPFC that occurred while a participant watched others play a stopwatch game. The connectivity between the two regions was greater when a participant watched a player win the game. In the present study, we found higher mu-band phase synchrony in a wide region of the mid-frontal electrodes when participants watched their cheered-for player win. This result indicates that the region under the C3 electrode was dynamically linked with a wide region of the mid-frontal electrode via mu synchronization during the outcome period. Furthermore, CFC analysis revealed higher synchronization between the mu and beta bands at F4 during the win trials. This mu-beta synchronization, which could be observed within a single electrode, indicates that integration of the functions underlying mu and beta band took place when participants watched their preferred player win the RPS game. This was also consistent with previous studies. Kawasaki et al. (2014) showed that frontal beta band (24 Hz) was coupled with theta band when participants received positive feedback during a visual monetary task, and also reported that frontal beta band was associated with the evaluation of the reward. In our study, phase synchronization indicated that the mu ERD observed at the motor area electrode (C3) was linked with F4 electrode, where we observed reward-related beta band power enhancement. Furthermore, cross-frequency coupling revealed integration between the linked alpha band which reflects MNS activity and the reward-related beta band. These results indicate that oscillations observed at the mid-frontal electrode interact with different functional oscillations to accomplish given cognitive functions. It is interesting that we found the coupling between the mu rhythm relating with the MNS and beta rhythm relating with the vicarious reward. In terms of social cognition, temporo-parietal junction (TPJ) has been reported to be involved in higher-level social cognitive functions. For example, Janowski et al. (2013) showed the functional connectivity between the VMPFC and the inferior parietal lobule in a task where the participant vicariously buys an item on behalf of another. On the other hand, MNS is considered to be lower-level social cognitive function. It is close to a motor representation and is an automatic reaction, such as simple action understanding or understanding of action goal (Caggiano et al., 2012). In our experiment, participants were just cheering for and observing the player. Then, we found the coupling between the mu rhythm relating with the MNS and beta rhythm relating with the vicarious reward. The result is consistent with the report of Shimada et al. (2016), which showed the connectivity between the MNS and the vmPFC by using fMRI and vicarious reward task. So, we consider that the vicarious reward processing is driven with the comparatively low-level social cognitive function.

Some researchers define vicarious reward as a positive feature of vicarious experience (Lockwood, 2016), and vicarious experience is thought to play a key role in empathy. An important distinction is often made between the emotional/affective and cognitive aspects of empathy. Previous studies have revealed that, compared with cognitive empathy, emotional empathy more strongly recruits neural circuits including the MNS (Nummenmaa et al., 2008; Shamay-Tsoory et al., 2009). Indeed, emotional empathy may induce a more realistic representation of an experience by recruiting the MNS. If recruiting the MNS is the key to inducing a more realistic emotion in observers, then it is reasonable to expect that vicarious rewards will induce higher phase synchronization with respect to the MNS and reward system when a “cheered” for player wins the RPS game. There are some limitations in generalizing our results. One limitation that must be considered is that the sample we used is small, and our sample also included mostly male participants. Although conclusive EEG data on vicarious reward gender differences are not yet available, we cannot exclude the possibilities that there are any differences between genders responding to the observed player. Second limitation is that, we couldn't measure how hard participants cheered for the player because of the experimental paradigm that it is still unclear that degree of how hard participants cheered for the player could affect to receiving vicarious reward. As previous study by Mobbs et al. (2009) showed the correlation between similarity, ventral ACC and VMPFC, degree of how hard participants cheer for the observed player could possibly affect to the vicarious reward receiving. Third limitation is that, we used scalp EEG recordings, which have an intrinsic limit in space resolution. We only found phase synchronization from the scalp EEG that intrinsic neural mechanism is still not clear. Although these ideas are purely hypothetical, they might be useful in guiding future research.

## Data Availability Statement

The datasets generated for this study are available on request to the corresponding author.

## Ethics Statement

The protocol was approved by the Research Ethics Committee of Meiji University, and conducted according to the principles and guidelines of the Declaration of Helsinki.

## Author Contributions

TI and SS: conceptualization. TI and TZ: data curation and formal analyses. TI, TZ, and SS: methodology. TI: writing—original draft. SS: writing—review and editing.

### Conflict of Interest

The authors declare that the research was conducted in the absence of any commercial or financial relationships that could be construed as a potential conflict of interest.
